# Testing a Communication Assessment Tool for Ethically Sensitive Scenarios: Protocol of a Validation Study

**DOI:** 10.2196/12039

**Published:** 2019-05-08

**Authors:** Thierry Daboval, Natalie Ward, Jordan R Schoenherr, Gregory P Moore, Caitlin Carew, Alicia Lambrinakos-Raymond, Emanuela Ferretti

**Affiliations:** 1 Department of Pediatrics Children's Hospital of Eastern Ontario University of Ottawa Ottawa, ON Canada; 2 Performance and Evaluation Genome Canada Ottawa, ON Canada; 3 Department of Psychology Carleton University Ottawa, ON Canada

**Keywords:** medical education, ethics, assessment tool, communication, neonatology

## Abstract

**Background:**

Although well-designed instruments to assess communication during medical interviews and complex encounters exist, assessment tools that differentiate between communication, empathy, decision-making, and moral judgment are needed to assess different aspects of communication during situations defined by ethical conflict. To address this need, we developed an assessment tool that differentiates competencies associated with practice in ethically challenging situations. The competencies are grouped into three distinct categories: communication skills, civility and respectful behavior, clinical and ethical judgment and decision-making.

**Objective:**

The overall objective of this project is to develop an assessment tool for ethically sensitive scenarios that measures the degree of respect for the attitudes and beliefs of patients and family members, the demands of clinical decision-making, and the success in dealing with ethical conflicts in the clinical context. In this article, we describe the research method we will use during the pilot-test study using the neonatal context to provide validity evidence to support the features of the Assessment Communication Tool for Ethics (ACT4Ethics) instrument.

**Methods:**

This study is part of a multiphase project designed according to modern validity principles including content, response process, internal structure, relation to other variables, and social consequences. The design considers threats to validity such as construct underrepresentation and factors exerting nonrandom influence on scores. This study consists of two primary steps: (1) train the raters in the use of the new tool and (2) pilot-test a simulation using an Objective Structured Clinical Examination. We aim to obtain a total of 90 independent assessments based on the performance of 30 trainees rated by 15 trained raters for analysis. A comparison of raters’ responses will allow us to compute a measure of interrater reliability. We will additionally compare the results of ACT4Ethics with another existing instrument.

**Results:**

This study will take approximately 18 months to complete and the results should be available by September 2019.

**Conclusions:**

ACT4Ethics should allow clinician-teachers to assess and monitor the development of competency of trainees’ judgments and communication skills when facing ethically sensitive clinical situations. The instrument will also guide the provision of meaningful feedback to ensure that trainees develop specific communication, empathy, decision-making, and ethical competencies.

**International Registered Report Identifier (IRRID):**

PRR1-10.2196/12039

## Introduction

### Importance of Ethics and Communication Education in Medicine

Clinicians face ethically challenging situations such as limiting care, engaging in collective or shared decision-making and surrogate decision-making, and participating in end-of-life discussions. In order to engage in successful clinical decision-making during these situations, specific competencies must be identified, education milestones should be established, and learning needs must be monitored. Given the complexity and ubiquity of these cases requiring tactful communication, high-stakes moral and ethical judgment, and mindfulness, training must start early during undergraduate and continue into postgraduate health care professional education [1-4]. The development of these competencies requires the promotion of skills associated with shared decision-making, increased empathetic accuracy of clinicians, and a focus on decreasing psychological distress for severely ill patients and their families [5-7].

Although all health care professions involve difficult situations, neonatology presents a diverse set of considerations including a wide range of ethically sensitive situations and frequent inter- and intradisciplinary communication. Supporting this, there is a growing recognition that Neonatal-Perinatal Medicine (NPM) trainees require a greater understanding of ethical features of clinical situations and that there is a need to increase the availability and efficacy of ethics and communication programs [8]. Our team implemented a neonatal ethics teaching program that integrates knowledge of ethics acquisition and provides opportunities to practice communication skills in a safe, structured environment [9]. This program helps develop ethical competencies by providing constructive feedback and promoting self-reflection [10]. Our neonatal ethics teaching program and other neonatal ethics published curricula focus on helping trainees refine their knowledge of ethics and learn competencies relevant to professionalism and communication [11,12]. While multiple approaches based on adult learning theory support trainees’ education, pedagogical methods best suited to teaching ethics and communication have yet to be identified. Assessment methods that can differentiate specific features of these situations need to be developed. These assessment tools can be used to support three important objectives: monitoring the development of a clinician’s knowledge and skills over the course of their career, supporting the evaluation of teaching methods, and guiding the design of effective education programs [13]. We considered these objectives in the development of our assessment tool.

### Need for a Communication Assessment Tool for Ethically Sensitive Clinical Situations

Studies focusing on NPM residency training have identified the need to develop tools supported by empirical evidence that assess knowledge and behavioral learning [8,14]. In addition to NPM, many other subspecialties in medicine need such assessment instruments. While knowledge tests in medical ethics competency and attitudes have been described for pediatrics and internal and neonatal medicine [15-18], they are not sufficient in and of themselves; assessment of general competencies such as communication skills must also be considered [9,11,19-21]. Our literature review identified several well-designed tools to assess communication skills during medical interviews. They were typically designed for medical encounters exploring symptoms or providing difficult news to patients and their family members. We failed to identify any that were directly related to medical ethical situations [22-27]. For instance, the Gap-Kalamazoo Communication Skills Assessment Form (GKCAS), which is used to assess communication competencies during complex encounters across medical subspecialties, includes domains such as building relationships, understanding the family perspective, sharing information, demonstrating empathy, and reaching agreement, but we believe it lacks important topics such as moral judgment and ethical conflicts [28].

### Foundation of the Communication Assessment Tool for Ethics Framework

Against this background, we created an assessment instrument designed to differentiate competencies related to ethical judgment and decision-making, respect and empathy, and communication skills during ethically sensitive situations. Although developed in the context of NPM, the Assessment of Communication Tool for Ethics (ACT4Ethics) can be applied to a wide range of ethically challenging situations encountered in many fields in the health care professions. This assessment instrument includes key milestones in the roles of medical professional, communicator, and collaborator [11]. It includes competencies aligned with verbal and nonverbal communication skills adapted to medical interviews [23]; communication skills included in delivering bad news [26], demonstrating awareness of ethical features of a situation including virtue ethics; bioethical principles and communicative ethics [29]; ethical judgment and decision-making [30]; and engaging patients in a decision-making process [31]. We used the academic literature to guide the construction of the ACT4Ethics scale. We grouped our assessment criteria into three broad domains reflecting a proposed set of distinct competencies: communication, civility and respectful behavior, and clinical and ethical judgment and decision-making.

The communication skills subscale evaluates basic verbal and nonverbal skills, while also addressing more complex skills such as conversational pragmatics that might arise during a clinical encounter [22,32]. Given the often ambiguous nature of the clinical setting as well as differences in clinicians’ and patients’ knowledge, this communication subscale also assesses whether a learner effectively closed the loop (ie, sought explicit confirmation that they shared an understanding) during critical periods throughout the encounter as well as at the end of the encounter [33]. Ethically sensitive scenarios regularly include elements such as presenting affectively charged information that would reasonably disappoint patients and requires the consideration of empathy [26] and respect for their values and beliefs.

The civility and empathy subscale assesses empathy-related skills and overt social cues associated with respect. Civility has become a growing concerning in professional environments [34,35] and clinical settings [36-38]. In contrast to respectful and disrespectful behaviors, incivility reflects actions wherein the intentions of the communicator lack clarity. This lack of clarity can lead to negative consequences over lengthy periods [34]. Similarly, empathy can be defined along two separate dimensions. Whereas cognitive empathy requires understanding another’s thoughts and beliefs in order to predict behavior [39], affective empathy involves sharing the emotional response for another’s joy and pain [40]. These appear to have a distinctive neurological basis [41,42]. Crucially, in the context of a clinical encounter, patients and family members might not disclose relevant information or consider clinical alternatives if they do not believe that clinicians respect their emotional responses, beliefs, or choices.

The judgment and decision-making subscale assesses clinical and ethical features of judgment and decision-making. Judgment and decision-making require consideration of a clinician’s awareness of the situation [43] and whether the clinician gathers and assesses evidence and considers diagnostic and treatment alternatives in an unbiased manner [44] given the constraints of the situation [45]. In the clinical context, this involves considering multiple sources of information such as that provided by other health care professionals, patients, and family members. Even if a clinician is an effective communicator and has demonstrated respect and empathy with a patient, failure to integrate the available information will ultimately undermine the clinical encounter.

### The Communication Assessment Tool for Ethics Scale

**Figure 1:**
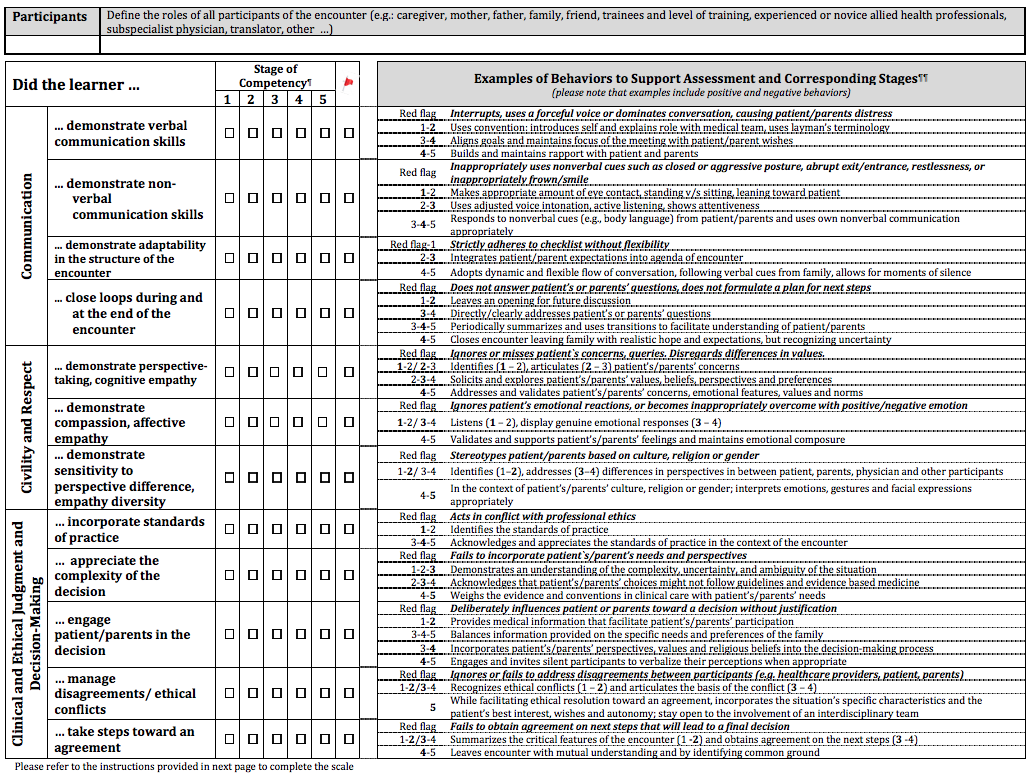
Assessment Communication Tool for Ethics (ACT4Ethics), page 1.

**Figure 2:**
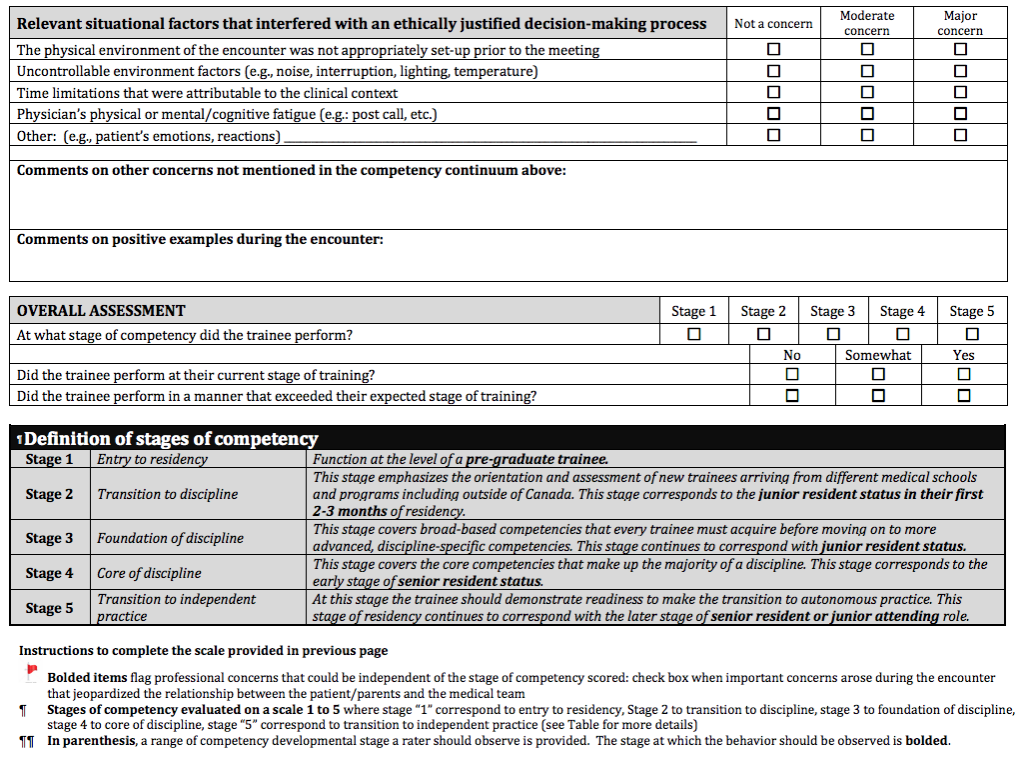
Assessment Communication Tool for Ethics (ACT4Ethics), page 2.

Figures 1 and 2 present the structure of the ACT4Ethics scale.

The ACT4Ethics scale includes 12 competencies aligned to the three domains included in the framework. The tool includes a 5-level rating scale for each competency with examples of milestones and stages in accordance with the continuum base by design model [11] to guide the rating. A red flag is added to the rating scale to allow the assessing clinicians to note concerns severe enough that a trainee should not currently be permitted to talk to patients or family members without supervision. The tool allows capturing the clinical context of the interaction and the relevant situational factors that might have justifiably interfered with the ethical judgment and decision-making processes. Such qualifications are necessary in order to account for any variability introduced by atypical features of clinical encounters likely to occur outside the simulation context. In the end, ACT4Ethics includes an overall assessment scale. Ideally, ratings on this overall scale should positively correlate with the averages obtained from the individual subscales.

The pilot study will present the tool without displaying the hypothesized targeted competencies depicted in subscale labels in order to ensure that raters will not be biased in assigning their ratings. For instance, while we have grouped certain items within a specific subscale, raters might assign ratings that do not support organizing ACT4Ethics in this manner.

### Aim of the Study

The objective of this study is to pilot-test ACT4Ethics. This study will be divided into two parts—part A: training the raters to use the tool and part B: obtaining validity evidence to support the scale construction [46].

## Methods

### Setting and Participants

The study will take place between February and June 2019 at the University of Ottawa Faculty of Medicine adjacent of the Children’s Hospital of Eastern Ontario (CHEO) and the Ottawa Hospital General Campus. CHEO is a tertiary pediatric center that houses a 3B neonatal intensive care unit, and the Ottawa Hospital General Campus is equipped with a birthing unit and a level 3A neonatal intensive care unit. The University of Ottawa Faculty of Medicine’s NPM training program is a 2- to 3-year residency accredited by the Royal College of Physicians and Surgeons of Canada.

The teaching tool used in the context of an Objective Structured Clinical Examination (OSCE) session is designed to test clinical skill performance that trainees in health care professions are expected to acquire [47]. The one-station OSCE will consist of a case focusing on antenatal findings of multiple congenital anomalies without a specific genetic syndrome identified. It will be divided into two parts for a total of 30 minutes. The first 15 minutes of the session will be videotaped and include one trainee and one standardized patient. One of the two coauthors (TD or EF) who directly observed the interaction will provide confidential formative feedback in the second 15 minutes of the session, which will not be videotaped.

The pilot group will consist of a purposive sample [48] of former and current trainees from the NPM, Maternal-Fetal Medicine, Obstetrics, Pediatric Palliative Care, Genetics, and General Pediatrics residency programs (n=30). Our recruitment strategy intends to obtain a wide distribution of performances to ensure that raters will use the full range of the scale. We will recruit and train academic staff (n=15) to review the videos of the pilot group. Standardized patients (n=2) will be recruited to play-act the patient in the OSCE.

### Ethical Considerations

Academic staff members from a variety of medical fields including collaborators who have participated in the creation of ACT4Ethics will also be piloting the tool. A research assistant or one of the authors (ALR) will approach academic staff, trainees, and standardized patients for consent. We will inform trainee participants that their choice will not affect their residency training assessment. Each academic staff participant will receive an incentive. The standardized patients, from the University of Ottawa Health Science Faculty, will be reimbursed for administration, training, and parking fees. The CHEO and Ottawa Health Science Network research ethics boards have approved this study.

### Study Design

This pilot cohort study is part of a multiphase project designed according to modern validity principles—content (phases 1 and 2: creation of a blueprint), response process (phase 3: pilot test), internal structure, relation to other variables, and consequences (phase 4: implementation) [46]—where evidence that supports a particular interpretation of the results is collected [49,50]. The design also considers threats to validity such as construct underrepresentation and factors exerting nonrandom influence on scores.

### Part A: Train the Raters

We will invite 15 academic staff members for training on ACT4Ethics and the GKCAS clinician/faculty form. A 45- to 60-minute video-based session developed by members of the project team (TD, EF, JS, and ALR) will be used to train academic staff/future raters on ACT4Ethics and help them achieve and promote consistency in assessment [51]. Future raters will test the tool on three short videos depicting a typical clinical scenario.

The short videos (approximatively 15 minutes in length) will be developed with the participation of one of the coauthors (ALR) acting out three levels of competence: transition to discipline, core of discipline, and advanced expertise [11], while interacting with a parent played by a standardized patient. A half-day session will involve at least three coauthors (TD, EF, ALR) or collaborators defining and standardizing the script for the videos. Each video will correspond to different levels of competence. They will include a number of key decision points where future raters should be able to identify and address specific features of a situation concerning communication (eg, ambiguous statements that require closing the loop), decision-making (eg, essential diagnostic information not presented), and respect/empathy (eg, family behavior or personal/religious beliefs affecting interaction).

### Part B: Obtain Validity Evidence

The pilot-test will be conducted using 30 distinct 15-minute videotaped interactions between a trainee and a standardized patient. We will ask the trainees to provide information on their previous experience in ethical encounters in their practice and ethical training, level of confidence in navigating an ethically charged situation, and basic demographics (eg, gender, age, year, and subspecialty of training). We will also ask them to complete an evaluation survey of the session.

Each trained rater will receive the videos of 6 different encounters, and they will use ACT4Ethics and GKCAS clinician/faculty form [27] to score trainee performance. The scores and debriefing will not be included in current trainees’ final subspecialty training evaluation. After providing their ratings, trained raters will provide feedback on a standardized satisfaction survey. Their feedback will allow comparison of rater views between ACT4Ethics and the GKCAS clinician/faculty form.

### Data Analysis

The research assistant or coauthor will enter all data into SPSS Statistics (IBM Corp). Descriptive statistics (mean and standard deviation or median and interquartile range) will be used to characterize participant demographics, responses to the survey, and the scores obtained with ACT4Ethics and compare them to the GKCAS clinician/faculty form.

Trainees’ prior experience with regard to ethical situations will allow us to measure its effect on their performance and interaction with the standardized patient, as well as on their overall scores from the ACT4Ethics and GKCAS tools.

To compare the scales, we will examine (1) the order of individual participants in terms of their aggregate performance for each scale and (2) the correlation between items from each subscale that should show a high degree of correspondence (eg, items that assess empathy in the civility and respect subscale). We anticipate that the trained raters will use the complete range of response scale and have their ratings correspond to the issues presented in the training videos. Each rater will assess a total of 6 videos to determine the interrater reliability of ACT4Ethics. This process will result in 90 independent evaluations assessed by 15 trained raters with each encounter being evaluated three times—three nested scores within participants. We will conduct a generalizability analysis with rater nested within video and crossed with item. This analysis will allow us to determine the proportion of variance accounted for by each variable as well as allowing us to generate reliability coefficients related to both interrater reliability and the internal consistency of the items on the scale. We will use the conventional level of reliability of .8 or higher for the overall subscale to analyze our results. We will additionally obtain correlations of ratings for items between subscales and within a subscale. The scores from raters using both assessment instruments will allow an evaluation of the correlation between ratings.

Responses from the satisfaction survey completed by the trained raters will be used to assess the acceptance and usability of ACT4Ethics and explore facilitators and barriers for future implementation. We will review feedback on the experience and on the tool to adapt ACT4Ethics as appropriate.

### Implementation Phase

The team will seek funding first to support the translation of ACT4Ethics into an electronic format ready for the one45 software platform, which supports the performance assessment of learners [52], and second, to facilitate the implementation of the tool in NPM, Pediatric, Maternal-Fetal Medicine, Medical Genetics, and Pediatric Palliative Care residency training programs. During this phase, the coauthors will continuously examine the construct validity of the assessment instrument.

## Results

We anticipate this project will take a total of 18 months to complete and expect the results to be available by September 2019.

## Discussion

### Assessment of Communication Tool for Ethics to Maximize Learning of Communication Skills

Engaging in conversations with patients and families facing ethically challenging situations requires well-trained staff with advanced communication skills to support them. These skills also facilitate shared decision-making about the provision of care that is in the patient’s best interest [53,54]. Physicians need to be taught these skills during their training. Without a well-designed assessment instrument specifically adapted to ethically sensitive situations, assessment of communication relies on subjectivity [55]. Such assessments will not maximize the learning of communication skills adapted to these encounters, leaving future physicians with underdeveloped skills potentially increasing the risk of conflict between patients, families, and the physician [56]. Although we are using an OSCE in the context of neonatology for the validation of our assessment instrument, the principles included in ACT4Ethics are also applicable in many other areas in health care professions. Our tool considers the specific affective, interconnected elements of verbal and nonverbal communication and cognitive features of ethically challenging clinical sets of circumstances encountered in medicine [24].

### Assessment Scale Construct Validity

ACT4Ethics was created through a multiphase process to develop a valid and reliable assessment tool [57]. Using an integrated knowledge translation model [58] to facilitate the implementation phase of ACT4Ethics into different medicine subspecialty residency programs, the project engaged key collaborators from different medical fields [49] with expertise in physician-patient communication, clinical ethics, and/or medical education. They participated in focus groups, dyadic interviews, and Web-based surveys to define the goals, use, and content of the assessment tool.

The instrument was developed to be (1) comprehensive, (2) easily applied within the clinical context, and (3) capable of adapting to many subspecialties across the health care professions while providing expedient assessment. The overall goal of ACT4Ethics is to guide specific, meaningful feedback to facilitate trainee identification of the ethical affordances of communication regarding complex clinical situations that arise in NPM and many medical subspecialties.

During scale development, collaborators identified components of unique competencies that would enable clinicians to balance the demands of rigorous clinical decision-making with the need to ensure that they address the ethically sensitive features of these scenarios. While related, we think that each of the 12 competencies used within the scale should allow us to examine how they might independently contribute to a successful clinical encounter. As an important determinant of the construct validity of an assessment instrument [46], each of the three subscales should assess distinct features of a clinical scenario and show minimal overlap in terms of the ratings. For instance, items on ACT4Ethics that assess a learner’s civility and respectful behavior should only show a weak relationship with items related to clinical judgment and communication. Ratings for items within individual subscales (eg, all communication items) should correlate with each other. Differentiating responses for items within one subscale from those in a different subscale will validate the internal structure of ACT4Ethics. Ratings should increase following a training session in a pre- and posttest design. Such a pattern would suggest that the scale captures the response processes associated with trainee acquisition of these skills [46].

Our study will attempt to demonstrate how ACT4Ethics is an improvement over other communication assessment scales. For instance, while ACT4Ethics and GKCAS [28] both assess empathy, our scale breaks down empathy in cognitive (eg, perspective-taking) [39] and affective (eg, feelings) [40] components, whereas GKCAS does not make this distinction. These constructs reflect an important distinction that is likely to be relevant in clinical practice: while a clinician might be able to understand a family member’s concerns, they might not be adequately emotionally responsive. Similarly, while GKCAS addresses relationship building, this construct is somewhat ambiguous. We instead assume that relationship building should be assessed by looking at the clinician’s ability to adapt, demonstrate sensitivity to differences in perspective by different individuals within a clinical encounter, engage family members in the decision, and manage disagreement and ethical conflicts. Our analyses will examine how ACT4Ethics and GKCAS relate to one another.

The logic and thoroughness behind the creation of ACT4Ethics support our belief that it will improve the assessment of trainees and positively contribute to providing objective, meaningful feedback on communication skills during challenging ethical situations. Nonspecific in-training rotation evaluations and other tools lack this type of feedback. By assessing competencies associated with communication skills, civility and empathy, along with clinical and ethical judgment and decision-making, we should be able to identify specific strengths and weaknesses of a particular trainee. Feedback from the ACT4Ethics scale can then be used by learners to promote self-reflection and strengthen their competencies in specific domains of practice. Overall, trends in ACT4Ethics scores of multiple trainees could allow program directors to readily identify teaching deficiencies and allow them to adjust their curriculum accordingly.

### Curriculum and Accreditation Needs

ACT4Ethics will assist with local curriculum and program development. We are currently evaluating the effect of our local neonatal ethics teaching program. Our evaluation follows Guskey’s levels of training program evaluation [13]. We have already evaluated reactions (Guskey level 1) and demonstrated that the neonatal ethics teaching program sessions were well-received by trainees and participants, with an overall satisfaction score of 5.8 out of 7 [2]. Currently, we are running our pre- and posttraining knowledge test with our ongoing cohorts to evaluate the acquisition of knowledge related to ethics (Guskey level 2) [18]. The support received from the University of Ottawa to implement the program and develop our tools and the expansion to other residency programs indicates that the organization supports our model (Guskey level 3: organization support and change). ACT4Ethics will allow our research team to evaluate learning and use of skills (Guskey level 4). For example, this tool may help to evaluate the efficacy of neonatal ethics teaching programs and other specific teaching strategies to improve learning and use of both knowledge and skills to navigate ethically challenging clinical situations. We believe that this tool can also provide other education and research teams with a means to evaluate the success of their educational interventions and programs.

The ACT4Ethics scale addresses a need identified by accreditation bodies. With an overarching goal of contributing to and improving the means through which the milestones included in the Royal College of Physicians and Surgeons Canada 2015 CanMEDS roles of communicator, professional, and collaborator are assessed, ACT4Ethics will allow mentors and supervisors to assess and monitor competency levels of trainees. Although the validation evidence will be obtained using the context of neonatology, the creation of videos depicting typical ethically sensitive scenarios encountered in other areas in health care professions can be used to demonstrate its generalizability. We anticipate that ACT4Ethics will guide clinician-teacher supervisors, making explicit which communication skills to assess and how to rate them according to a clear range of competence. The tool can provide trainees with a clear sense of what the expectations are for their communication skills during difficult and ethically sensitive conversations. Consistent and relevant feedback is more likely to affect trainees’ learning of skillful behaviors and communication skills [59], potentially improving patients’ and parents’ satisfaction relationship with the physician-trainee.

## Abbreviations

NPM: neonatal-perinatal medicine
GKCAS: Gap-Kalamazoo Communication Skills Assessment Form
ACT4Ethics: Assessment of Communication Tool for Ethics
CHEO: Children’s Hospital of Eastern Ontario
OSCE: Objective Structured Clinical Examination
